# Mandatory vaccination: understanding the common good in the midst of the global polio eradication campaign

**DOI:** 10.1186/s13584-017-0198-4

**Published:** 2018-01-03

**Authors:** Lawrence O. Gostin

**Affiliations:** O’Neill Chair in Global Health Law and Director, World Health Organization Collaborating Center on National and Global Health Law, Georgetown University, Washington, D.C, USA

**Keywords:** Polio, Public health law, Police powers, Common good, Autonomy

## Abstract

**Background:**

The detection of wild poliovirus in Israeli sewage in May 2013 led the health authorities to vaccinate children with OPV (Oral Polio Vaccine). Shelly Kamin-Friedman explored the legal and ethical dimensions of this policy. This commentary makes three claims: (1) Mandatory vaccination is a valid exercise of the state’s police powers to protect the common good. (2) A disease eradication campaign is a sufficient ground for the exercise of those powers. (3) The state is obliged to use the least restrictive/invasive measure to achieve community-wide vaccine coverage, but need not use less effective measures; further, determining which measure is most effective is a fact-specific determination.

**Goals:**

This commentary offers grounds to support state powers to protect the public’s health and safety. It shows why governments have both the duty and power to safeguard the collective good. State powers also have limits, whose boundaries are determined by the public health necessity. If the state is reasonably using the least restrictive intervention to achieve an important public health objective, it is well within the limits of its authority.

**Method:**

The commentary uses legal and ethical norms and evidence to support its conclusions.

**Main findings and conclusion:**

Governments have a duty and power to achieve population-based vaccine coverage sufficient to stem the spread of infectious diseases, including in isolated geographical areas with high numbers of individuals claiming religious and/or conscientious exemptions to vaccine requirements. Governments are obliged to reasonably seek the least restrictive/invasive measure to achieve valid public health objectives; and governments are not obliged to use less effective measures simply because they are voluntary or less invasive. Finding the most effective, least invasive intervention is fact-specific. The essence of public health law is to recognize the state’s power and duty to safeguard the public’s health and safety, and to establish and enforce limits on those powers when the government overreaches—that is, adopts a measure more invasive/restrictive than needed to achieve a valid public health objective.

Shelly Kamin-Friedman eruditely explores the ethical and legal dimensions of Israeli polio vaccination policies after authorities detected wild poliovirus in domestic sewage in May 2013 [1]. Her article raises several interrelated questions, which I will explore in turn: Is mandatory vaccination a valid exercise of the state’s police powers to protect the common good? Is disease eradication a sufficient ground for the exercise of these powers? And what are the least restrictive means to achieve the objective of community immunity from vaccine preventable infectious diseases?

I have little doubt that the police powers do grant public health authorities the power and duty to vaccinate the population. I also think there are strong reasons for including disease eradication as a sufficient justification for the exercise of those powers. As with all public health powers, government should always use the least invasive/restrictive alternative to achieve the public health objective. This does not require health authorities to use less effective measures, but if a less invasive intervention will achieve the public health goal as well, or better, it should be employed.

## The police powers and the common good

The linguistic and historical origins of the “police” powers demonstrate a close association between government and civilization: politia (the state), polis (city), and politeia (citizenship). “Police” was meant to describe those powers that permitted sovereign government to exercise authority to promote the common good, notably health and safety. “Police” had a secondary usage as well: cleansing or keeping clean, which resonates with public health connotations of hygiene and sanitation [2]. Vaccination is squarely within the police powers, as the most effective intervention to prevent the societal spread of infectious diseases. No individual acting alone can prevent the transmission of dangerous pathogens, which is why government has a special responsibility to safeguard the common good.

Compulsory immunization, of course, invades a personal sphere of autonomy and bodily integrity, but those rights are not absolute. In the vast majority of cases the societal benefits far outweigh the uncommon risks incurred by vaccinations. In very rare instances, the live attenuated vaccine-virus can genetically change into a form that can paralyze (known as a circulating vaccine-derived poliovirus or cVDPV). That is a real risk, but must be compared with the number of people saved through an effective vaccine strategy.

Vaccines, however, have historically spurred controversy, perhaps because they often require injection of a live attenuated virus in a person who is otherwise healthy [3]. Most vaccination campaigns are directed toward infants and children (and vaccine schedules recommend multiple immunizations in a short period during early childhood). The focus on dependent children has generated heightened concern among parents. When these characteristics of vaccination are combined with science skepticism and with ever-stronger claims of religious freedom, the risk of under-vaccination is real. Even when political communities achieve high overall vaccination compliance rates, infectious disease outbreaks occur in geographically and culturally/religiously isolated communities where parents claim exemptions from vaccination requirements.

Outbreaks of measles, for example, have occurred in many countries due to unwarranted fears of a link to autism [4]. The *Lancet*, which published the original account of a link between the Thimerosal (a mercury-based vaccine preservative) and autism, withdrew the article due to fraud; since that time a volume of scientific research has demonstrated no causal association [5]. The issue is whether vaccine refusals that have no scientific grounding should be respected when they lead to harmful consequences for the unvaccinated child, as well as others, particularly for vulnerable children who are immune-compromised. Mandatory vaccination, therefore, comes well within the “harm principle,” which justifies compulsion to prevent individuals from placing others at risk. Compulsion is also justified because it is a classic illustration of the “Tragedy of the Commons” [6]. Provided a sufficient percentage of the population is vaccinated, everyone benefits from community or “herd” immunity. But if enough people opt out of vaccinations, everyone is at heightened risk.

## Polio eradication as a ground for exercising the police powers

The Global Polio Eradication Initiative (GPEI), launched by a 1988 World Health Assembly resolution, aims for the complete eradication and containment of all wild, vaccine-related and Sabin polioviruses, such that no child ever again suffers paralytic poliomyelitis. The campaign has been highly effective, albeit costly, decreasing the global incidence of polio by 99.9%. GPEI estimates that 16 million people today are walking who would otherwise be paralyzed, and > 1.5 million people are alive, whose lives would have been lost [7]. But there have been pockets of disease that have been extraordinarily difficult to penetrate, often because governments are unstable or conspiracy theories have buttressed resistance in certain communities. In 2016, 37 cases of wild poliovirus were reported in Afghanistan, Nigeria, and Pakistan. War-torn countries, such as the Democratic Republic of the Congo and Syria, pose ongoing risks [8].

Is a global eradication campaign such as GPEI a sufficient justification for the exercise of police powers? The answer is yes, and for much the same reasons as for mandatory childhood vaccinations. When individuals refuse polio vaccination – even OPV (oral poliovirus vaccine) which causes vaccine-induced polio in rare cases – that refusal places his or her community at risk. But in the case of eradication campaigns, refusals can be even more consequential because they perpetuate a threat that would otherwise be eliminated with the successful culmination of the campaign. There is also another dimension of the tragedy of the commons in the context of disease ratification campaigns. Most countries and regions have eliminated wild polio, but so long as isolated communities refuse to cooperate with the global campaign, everywhere is at risk—especially given mass travel across countries and continents.

Refusers also cost money as the campaign is continually needing additional resources. These resources are taken from other global health priorities, at WHO and elsewhere. Polio eradication accounts for $902.8 million, 20% of WHO’s 2018–2019 budget [9]. It is in everyone’s interests, therefore, to move as quickly as possible to full polio eradication, both to eliminate the threat of an ancient scourge and to conserve global health resources.

There is also an international legal obligation to fight polio with effective vaccine campaigns. The International Health Regulations (IHR) is a World Health Organization treaty, binding on states parties [10]. On 5 May 2014, the World Health Organization Director-General (D-G) declared the international spread of poliovirus a Public Health Emergency of International Concern (PHEIC) under the IHR [2005]. The D-G issued Temporary Recommendations under the IHR to reduce the international spread, and requested a reassessment of wild polio outbreaks by the Emergency Committee every 3 months. The 15th meeting of the Emergency Committee was held in November 2017, continuing the state of emergency [11]. To comply with the Temporary Recommendations, any country infected by poliovirus should declare the outbreak as a national public health emergency. While Israel had not reported wild polio cases, the reservoir of virus in its sewage system posed a risk to its own population and beyond [12].

## Getting to vaccine compliance: mandates, what kinds, or other less restrictive measures

Two public health goals are intertwined: preventing wild polio virus outbreaks and ultimately eradicating the disease. Achieving those goals requires widespread compliance with vaccination campaigns, not only countrywide, but also among geographically isolated communities where refusers can fuel outbreaks. Coercion can be necessary to overcome popular resistance to vaccination, or it can be counter-productive—for example, by reinforcing fears of adverse effects or government overreach.

Facts on the ground – social, political, and cultural – should determine the best way to achieve high compliance. Thus, while sovereign states have the power to vaccinate as part of the global eradication campaign, they should utilize compulsion only if it would be more effective than voluntary or less restrictive measures. These are fact-based determinations without an a priori answer as to *which* legal measure should be employed.

There is an active debate in public health circles on the merits of vaccine mandates. Virtually all public health experts agree on the overall goal, which is to achieve uniformly high vaccination rates, including in areas with a high concentration of skeptics. But there are wide variations on how to achieve the objective: some scholars would opt directly to compulsory vaccination, while others would simply make it harder for individuals to refuse. Compulsory vaccination can be enforced using a variety of penalties, ranging from criminal sanctions and civil fines to denial of some public benefit, such as entry to school or childcare or public payments or tax credits for dependent children.

Vaccination mandates also can be structured in multiple ways, with no non-medical exemptions (“eliminationism”) or exemptions based on religious beliefs and/or conscience. The broadest exemptions allow virtually all religious or conscientious objectors to opt out of vaccine mandates. Narrow exemptions would require a genuine religious reason to opt out. The law could also make it burdensome to obtain an exemption, for example, by requiring claimants to fill in a detailed form and/or requiring attendance to immunization education sessions (so-called “inconvenience”) [13]. This approach raises questions of fairness, as better educated individuals would be in a better position to incur the burden. To reduce unfairness, some call for a “contribution” as a quid pro quo for opting out—for example, preparing healthy school meals or fundraising for charities [14].

## Conclusion

It is not necessary to decide here which method is most effective and fairest, or which is the least restrictive. I have my doubts about requiring highly unusual demands, such as education classes or services in kind, and would prefer to eliminate non-medical exemptions or design a narrow religious exemption that requires completion of an application, as we do for other kinds of exemption. The legal and ethical point is simply this. Governments have: (1) a *duty* to reduce infectious disease threats for the common good; (2) the *power* to compel vaccinations to achieve that public good; and (3) *limits* on that power, requiring a good faith exploration of equally effective, less restrictive alternatives. That is the essence of public health law properly considered—the state’s duty to safeguard the public, the power to achieve that public good, and the necessary limits under the rule of law [2].
